# Energy and Exhaustion May Explain Different Subdomains of Perceived Fatigability

**DOI:** 10.1093/geroni/igab046.1432

**Published:** 2021-12-17

**Authors:** Rain Katz, Rebecca Cohen, Theresa Gmelin, Kyle Moored, Yujia (Susanna) Qiao, Maggie Slavin, Nancy W Glynn

**Affiliations:** 1 University of Pittsburgh Graduate School of Public Health, Pittsburgh, Pennsylvania, United States; 2 University of Pittsburgh, Pittsburgh, Pennsylvania, United States; 3 University of Pittsburgh, University of Pittsburgh, Pennsylvania, United States

## Abstract

Fatigability is a more sensitive measure of one’s perception of fatigue. To identify an appropriate fatigue question when a fatigability measure is unavailable, we examined associations between widely used global fatigue questions and perceived physical and mental fatigability. Participants (N=896, age=74.7±6.6, 58.1% women) from two aging research registries completed the valid Pittsburgh Fatigability Scale (PFS, 0-50) and five global fatigue questions: energy level (0-10), running out of energy (0-5), feeling energetic (0-6), feeling tired (0-6), and feeling exhausted (0-6) over past four weeks. All fatigue measures were correlated (p<0.0001) with physical (|r| range=0.48-0.57) and mental fatigability (|r| range=0.31-0.39). “Energy level” and “feeling exhausted” had strongest associations with physical and mental fatigability, respectively, in age, sex, BMI-adjusted regression models (p’s<0.001), suggesting older adults can distinguish between physical and mental domains. Future work will explore how these constructs are distinct but related, and confirm the optimal proxy for the two fatigability subdomains.

